# Supplementary figures and images for: Plasmodium vinckei genomes provide insights into the pan-genome and evolution of rodent malaria parasites
Source: BMC Biol. 2021 Apr 23;19:69. doi: 10.1186/s12915-021-00995-5 (PMC8063448; doi:10.1186/s12915-021-00995-5)

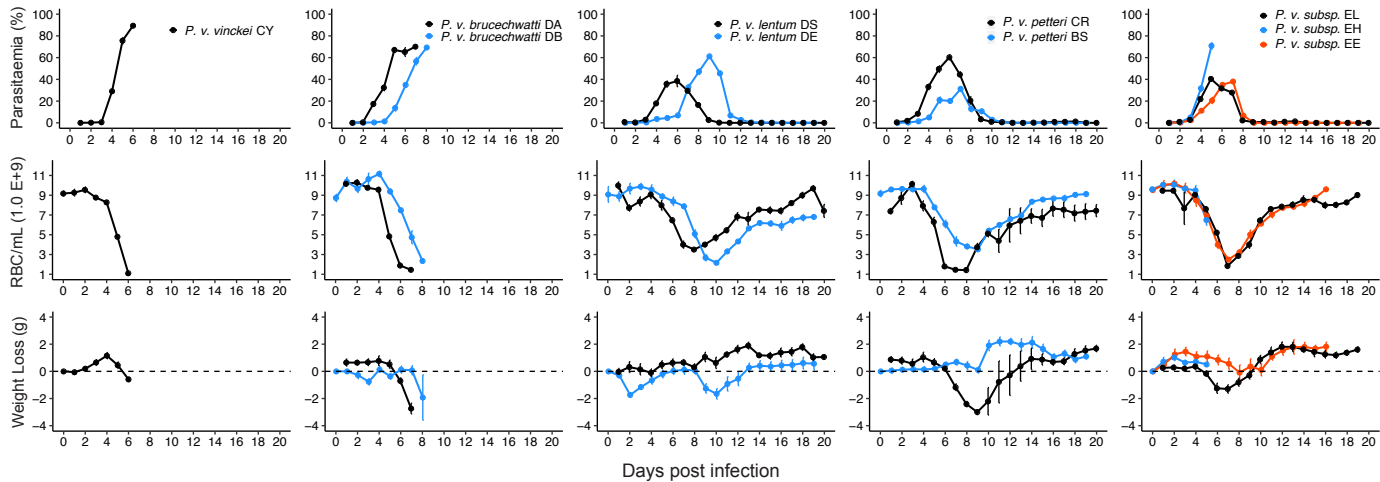

Supplement: Supplementary file 2 — Additional file 2. Infection profiles of ten Plasmodium vinckei isolates. Changes in parasitaemia, host RBC density and host weight during P. vinckei infections. Error bars show standard deviation of the readings within five biological replicates. † denotes host mortality [file 12915_2021_995_MOESM2_ESM.pdf]

A

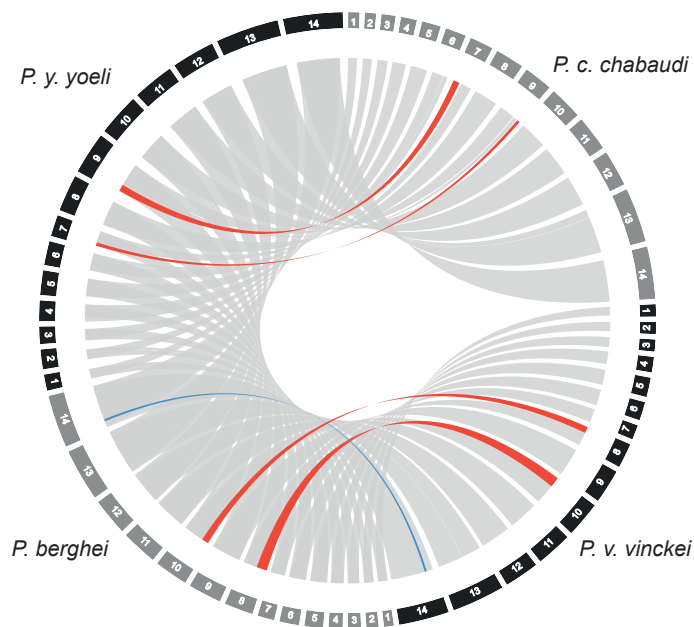

C

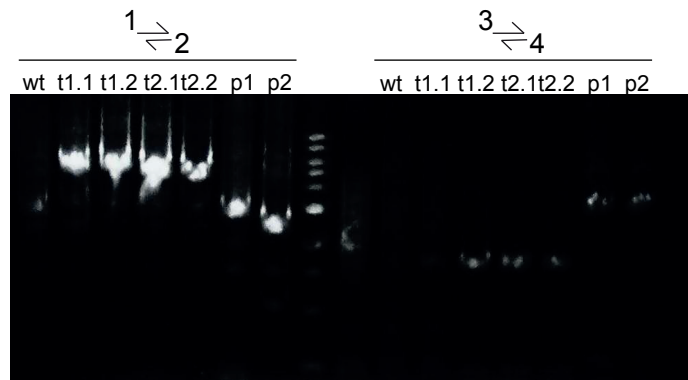

B

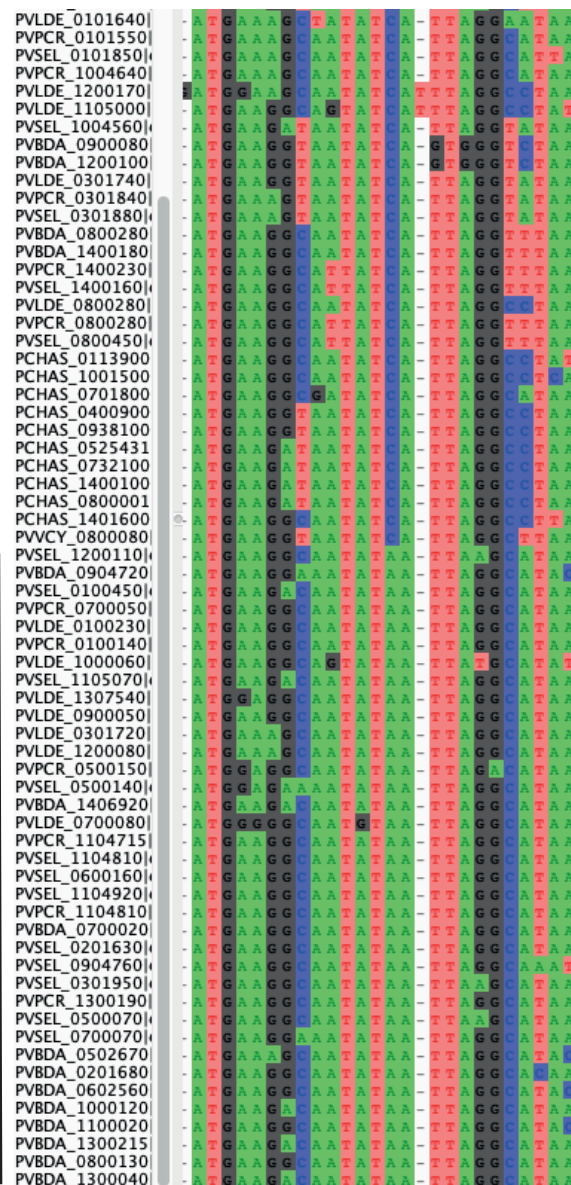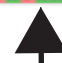

Supplement: Supplementary file 12 — Additional file 12. A) Circos figure showing rearrangements among four RMP species B) Gene alignment of pseudogenised ema1 genes. C) PCR bands showing successful integration of the pPvvCY-p230p-gfpLuc plasmid (two clones, p1 and p2) in the p230p locus at the 5′ (primers 1–2) and 3′ (primers 3–4) ends. Bands of expected sizes (5791 bps at the 5′ end and 1629 bps for 3′ end) were amplified from genomic DNA of transfectant parasites from two independent transfections (t1.1 and t1.2 for p1; t2.1 and t2.2 for p2). No band was obtained for wildtype control (wt) and non-specific bands were obtained for integration plasmids (p1 and p2) used as episomal controls [file 12915_2021_995_MOESM12_ESM.pdf]
