# Supplementary material for: Plasmodium vinckei genomes provide insights into the pan-genome and evolution of rodent malaria parasites
Source: BMC Biol. 2021 Apr 23;19:69. doi: 10.1186/s12915-021-00995-5 (PMC8063448; doi:10.1186/s12915-021-00995-5)

Re-evaluation of *pir, fam-a and fam-b* multigene families

For *pirs*, we obtained four long-form and eight short-form clades as in [26] (tree 10) albeit with lower bootstrap support, possibly due to our overly stringent automated trimming of the sequence alignment (see Methods). With a few exceptions, *P. vinckei pir* genes majorly populated two clades - L1 and S7 and a subclade S1g. These clades, previously shown to be *P. chabaudi*-dominant, hold equal or near equal proportions of *P. vinckei* pirs too. The only other *P. chabaudi*-dominant clade, L4, remains as a completely *P. chabaudi*-specific gene expansion. No *P. vinckei* species- or subspecies-specific clades are evident except for two subclades that could be inferred as *Pvv*CY-specific expansions within L1 and S7 (marked i and ii in tree 10). Speciation of *P. vinckei* subspecies from their common ancestor seems to have been accompanied by gene gain in L1 and S7 clades and gene loss in S1g subclade. There is an almost linear increase of around 20 genes in *Pvv*CY, *Pvb*DA and *Pvl*DE in clade L1 pirs and a near doubling of clade S7 pirs in *Pvp*CR.

The *fam-a* and *fam-b* phylogenies (tree 3 and 4 respectively) show that previously identified ancestral lineages [25] are maintained in *P. vinckei* too. The addition of *P. vinckei* genes resolved the ancestral clade of internal *fam-a* genes in chromosome 13 further into several well-supported *vinckei* group clades and a *berghei* group clade (marked as A in tree 3). The 19 other *fam-a* clades and five *fam-b* clades consisting of positionally conserved orthologous genes are also conserved in *P. vinckei* (pan-RMP clades marked with * in tree 3 and 4). The *fam-a* family has expanded in the non-Katangan *P. vinckei* subspecies through independent events of gene duplication in their common ancestor giving rise to several non-Katangan clades (marked as B in tree 3). There is only a moderate *P. vinckei*-specific expansion in *fam-b* giving rise to three clades (marked as A in tree 4) that includes *Pvv*CY genes too, pointing to gene

duplications in the *P. vinckei* common ancestor. In both the phylogenies, species and subspecies-specific gene duplication events within the *vinckei* group are rare but do occur (marked as i-iv in tree 3 and 4).

fam-d **multigene family**

The *fam-d* multigene family is present as a single ancestral copy in *P. berghei* internally on chromosome IX but is expanded into a gene cluster in the same loci in *P. yoelii* (5 genes) and *P. chabaudi* (21 genes). Similar expansions have occurred in *P. vinckei* subspecies and phylogenetic analysis shows the presence of six robust clades within this family (tree 6). Clade I is clearly the ancestral clade from which all other *fam-d* genes have been derived as it consists of the single *P. berghei* gene and its orthologs in other RMPs, positionally conserved to be the outermost gene of the *fam-d* cluster in each RMP. While the *fam-d* family in *P. yoelii* is completely a product of paralogous expansion within Clade I, the *fam-d* families in the *vinckei* group seem to have expanded *via* five ancestral lineages forming clades II-VI. A subset of orthologs in Clade II (marked with * in tree 6) are positionally conserved among *vinckei* group parasites, located immediately after the *fam-d* ancestral copy and could therefore represent the Clade II ancestral gene in the *vinckei* group common ancestor. *Pvv*CY has a smaller *fam-d* repertoire of 6 genes derived from only three of the five *vinckei* group lineages (Clade II, IV and VI), apart from the conserved ancestral copy.

Other multigene families

ML-based trees for haloacid dehalogenase-like hydrolase (*hdh*), putative reticulocyte binding proteins (*p235*) and lysophospholipases (*lpl*) have generally well-resolved topologies with robust bootstrap support for their nodes and some clades contain

syntenic orthologous genes (clades marked with * in trees 7, 8 and 9) to member genes in *P. falciparum,* for example, *Pf*HAD2, *Pf*HAD3, *Pf*HAD4 and *Pf*RH6. Poor bootstrap support was obtained for the *etramp* tree (tree 2), however clades were identified for some members including *uis3*, *uis4* and *etramp10.2*.

1. erythrocyte membrane antigen 1 (*ema1*)

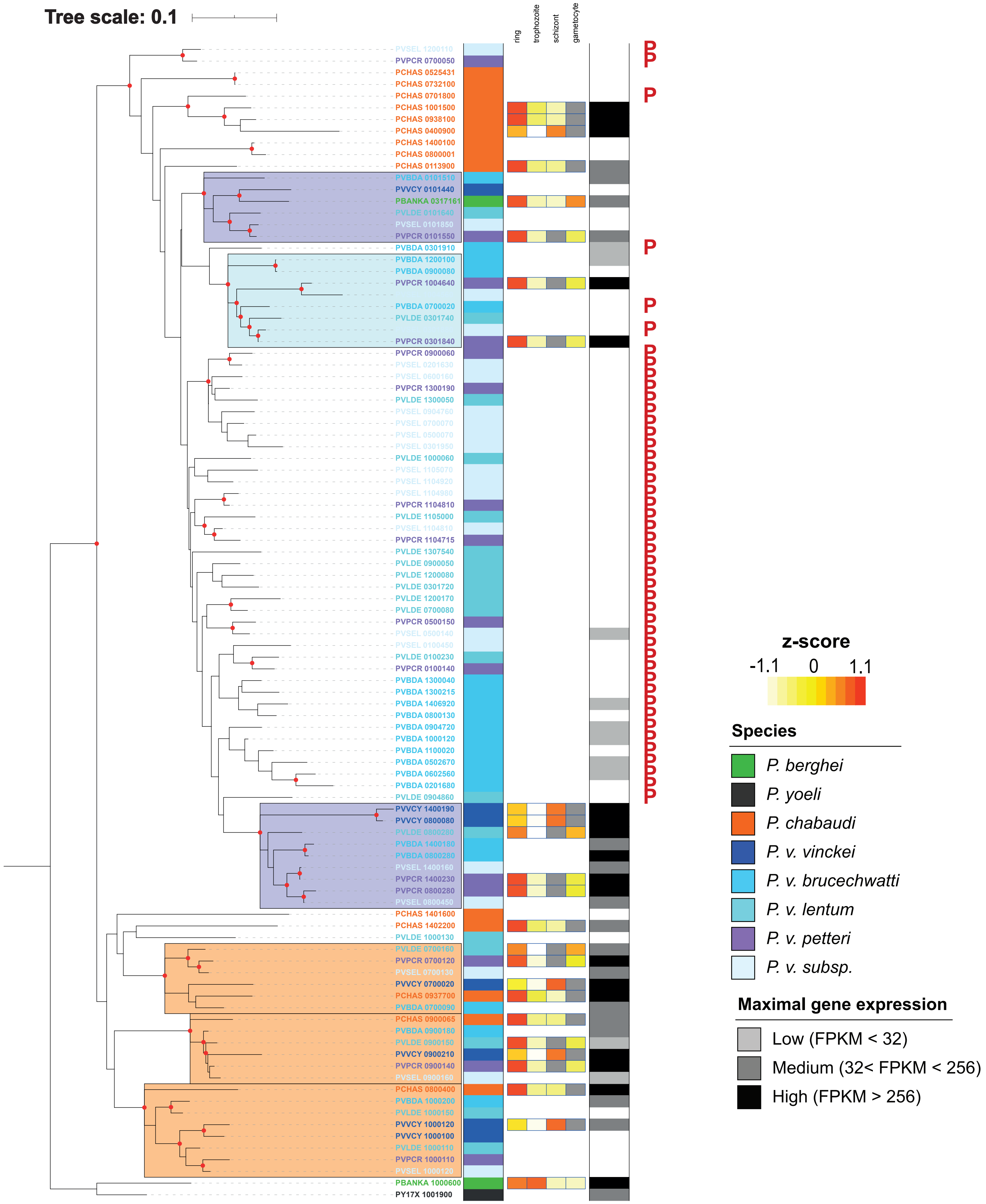

2. early transcribed membrane protein (*etramp*)

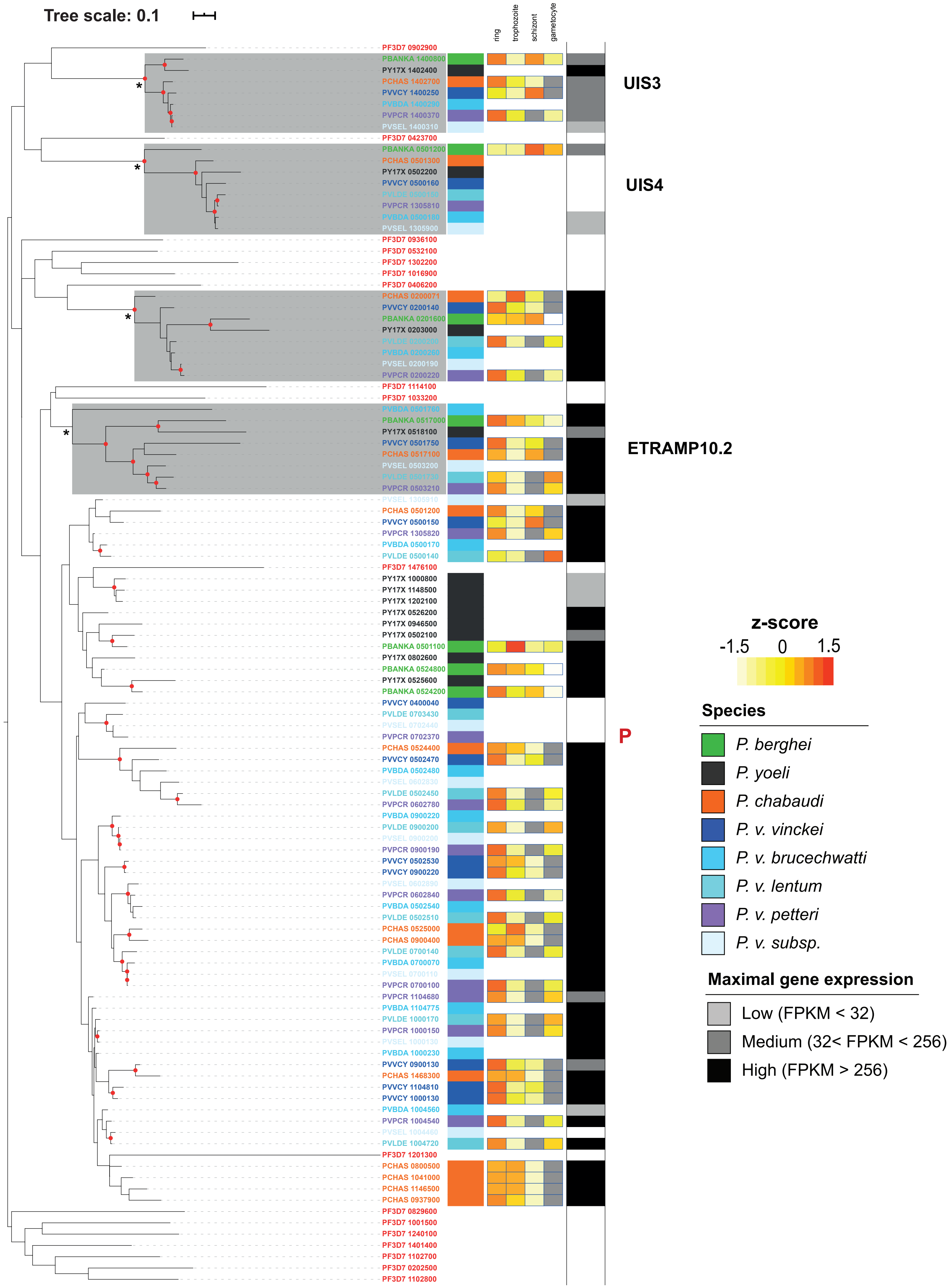

3. RMP-*fam-a*

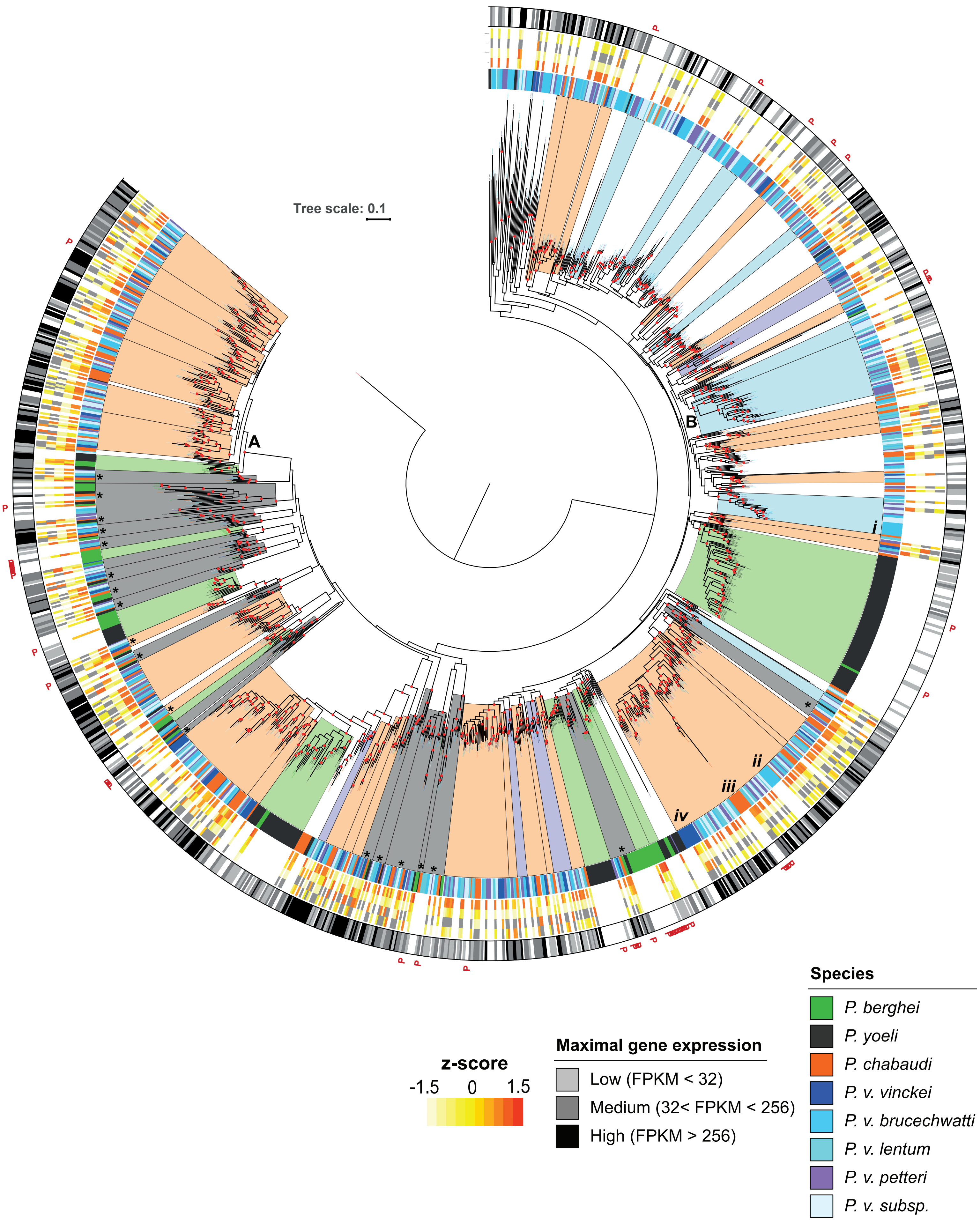

Tree scale: 0.1
A
B
i
ii
iii
iv
z-score
-1.5
0
1.5
Maximal gene expression
Low (FPKM < 32)
Medium (32< FPKM < 256)
High (FPKM > 256)
Species
P. berghei
P. yoeli
P. chabaudi
P. v. vinckei
P. v. brucechwatti
P. v. lentum
P. v. petteri
P. v. subsp.

4. RMP-*fam-b*

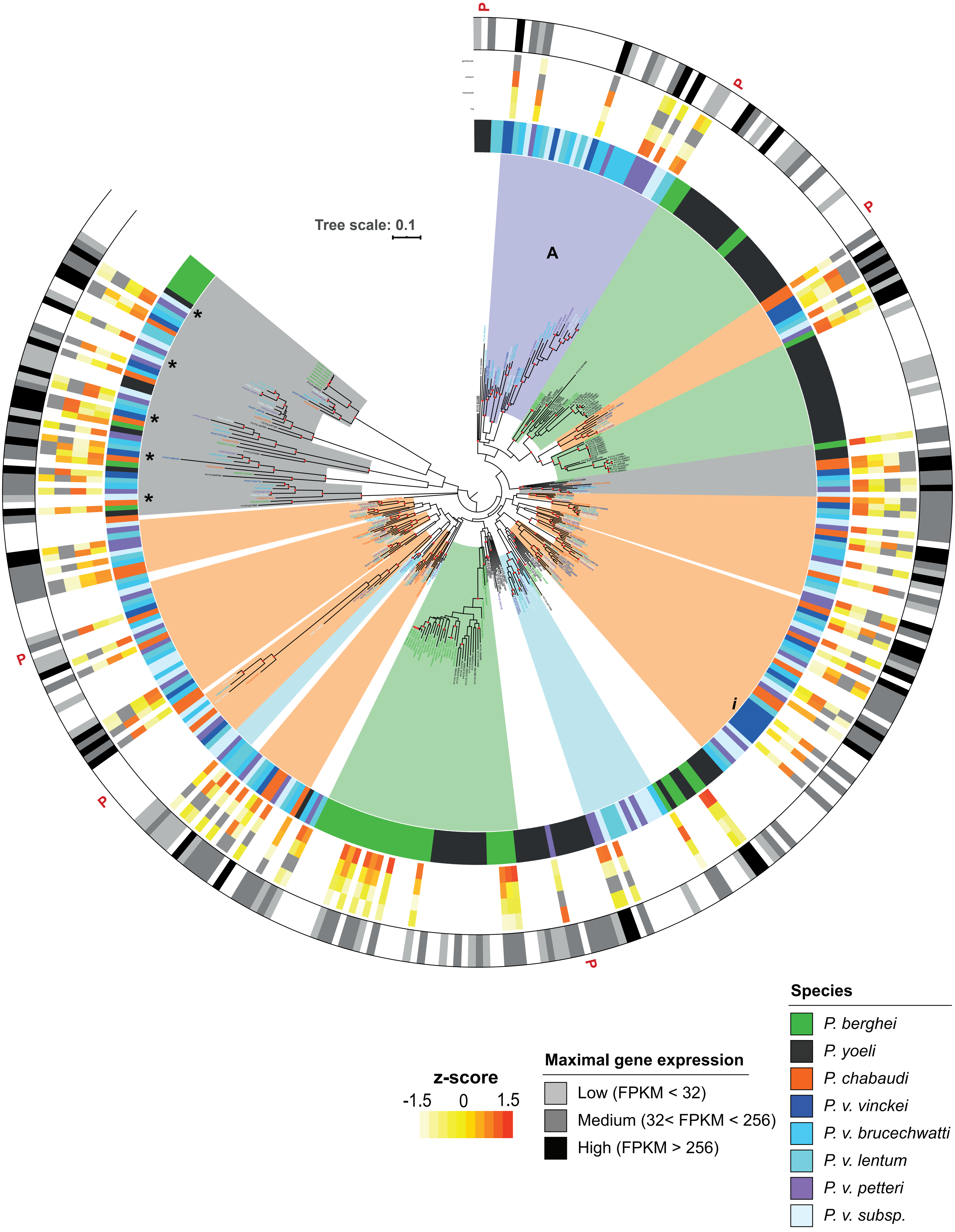

Tree scale: 0.1
A
i
*
P
gametocyte
schizont
trophozoite
ring
z-score
-1.5
0
1.5
Maximal gene expression
Low (FPKM < 32)
Medium (32< FPKM < 256)
High (FPKM > 256)
Species
P. berghei
P. yoeli
P. chabaudi
P. v. vinckei
P. v. brucechwatti
P. v. lentum
P. v. petteri
P. v. subsp.

5. RMP-*fam-c*

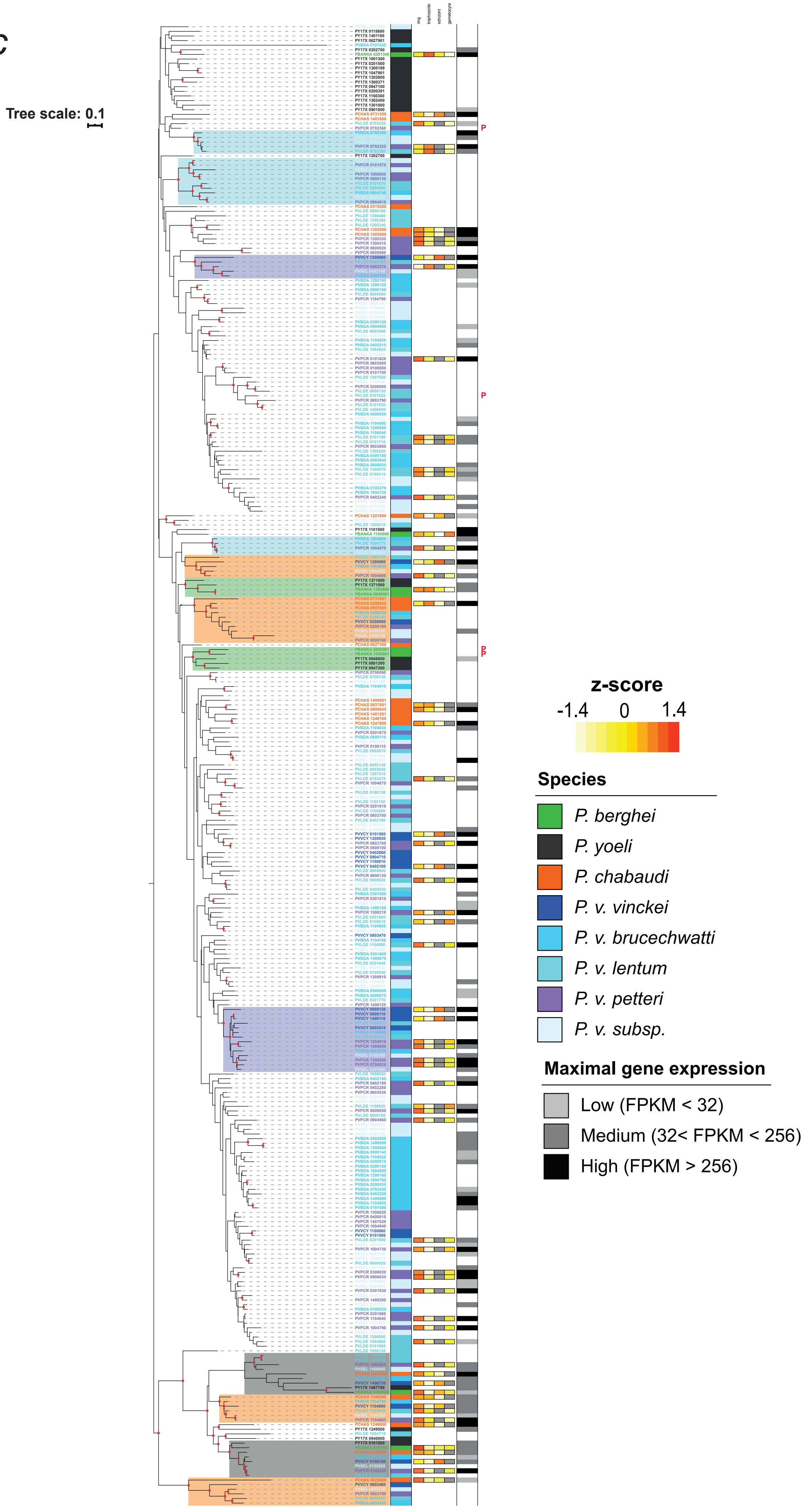

Tree scale: 0.1
z-score
-1.4
0
1.4
Species
P. berghei
P. yoeli
P. chabaudi
P. v. vinckei
P. v. brucechwatti
P. v. lentum
P. v. petteri
P. v. subsp.
Maximal gene expression
Low (FPKM < 32)
Medium (32< FPKM < 256)
High (FPKM > 256)

6. RMP-*fam-d*

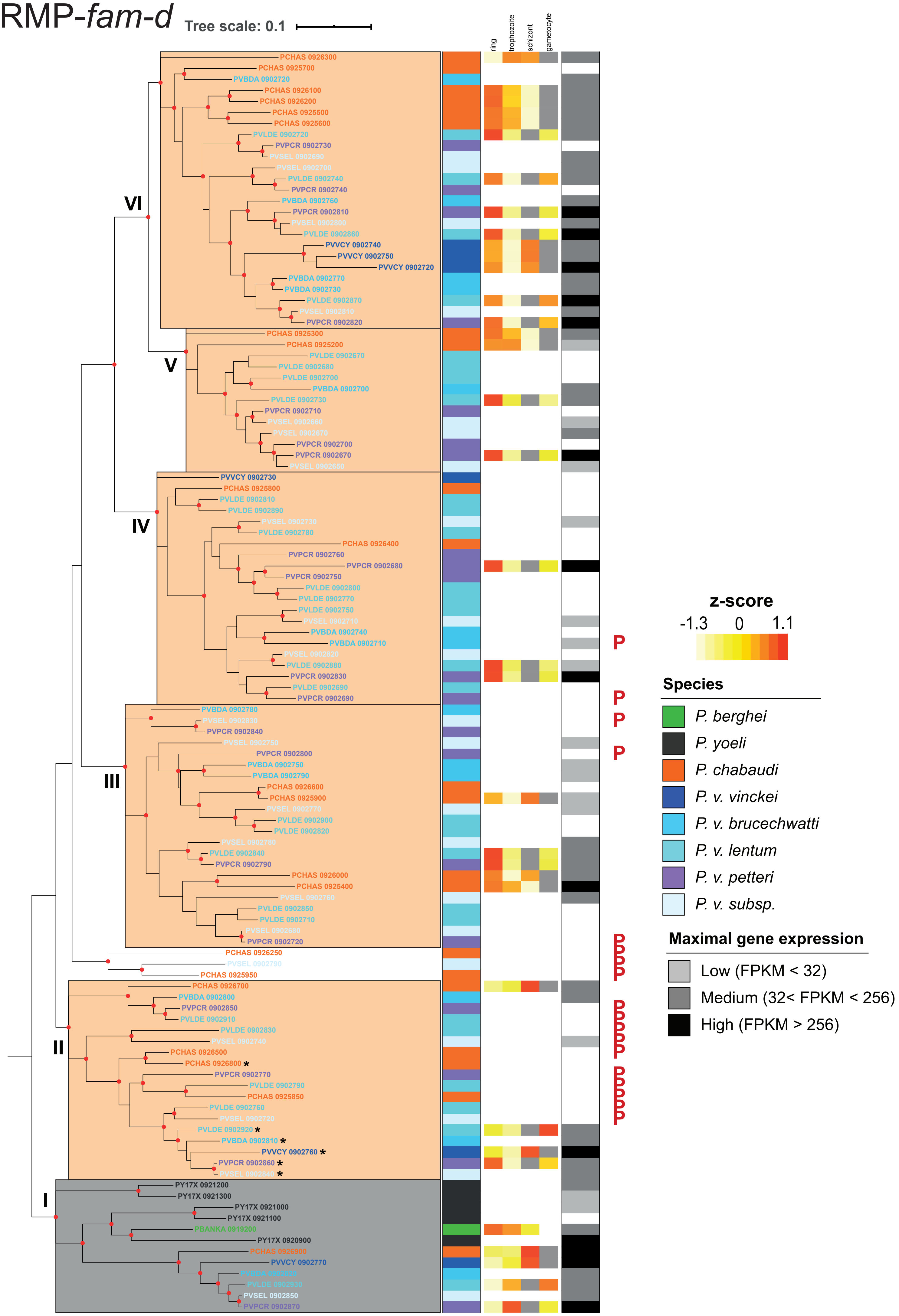

Tree scale: 0.1
ring
trophozoite
schizont
gametocyte
VI
V
IV
III
II
I
PCHAS 0926300
PCHAS 0925700
PVBDA 0902720
PCHAS 0926100
PCHAS 0926200
PCHAS 0925500
PCHAS 0925600
PVLDE 0902720
PVPCR 0902730
PVSEL 0902690
PVSEL 0902700
PVLDE 0902740
PVPCR 0902740
PVBDA 0902760
PVPCR 0902810
PVSEL 0902800
PVLDE 0902860
PVVCY 0902740
PVVCY 0902750
PVVCY 0902720
PVBDA 0902770
PVBDA 0902730
PVLDE 0902870
PVSEL 0902810
PVPCR 0902820
PCHAS 0925300
PCHAS 0925200
PVLDE 0902670
PVLDE 0902680
PVLDE 0902700
PVBDA 0902700
PVLDE 0902730
PVPCR 0902710
PVSEL 0902660
PVSEL 0902670
PVPCR 0902700
PVPCR 0902670
PVSEL 0902650
PVVCY 0902730
PCHAS 0925800
PVLDE 0902810
PVLDE 0902890
PVSEL 0902730
PVLDE 0902780
PCHAS 0926400
PVPCR 0902760
PVPCR 0902680
PVPCR 0902750
PVLDE 0902800
PVLDE 0902770
PVLDE 0902750
PVSEL 0902710
PVBDA 0902740
PVBDA 0902710
PVSEL 0902820
PVLDE 0902880
PVPCR 0902830
PVLDE 0902690
PVPCR 0902690
PVBDA 0902780
PVSEL 0902830
PVPCR 0902840
PVSEL 0902750
PVPCR 0902800
PVBDA 0902750
PVBDA 0902790
PCHAS 0926600
PCHAS 0925900
PVSEL 0902770
PVLDE 0902900
PVLDE 0902820
PVSEL 0902780
PVLDE 0902840
PVPCR 0902790
PCHAS 0926000
PCHAS 0925400
PVSEL 0902760
PVLDE 0902850
PVLDE 0902710
PVSEL 0902680
PVPCR 0902720
PCHAS 0926250
PVSEL 0902790
PCHAS 0925950
PCHAS 0926700
PVBDA 0902800
PVPCR 0902850
PVLDE 0902910
PVLDE 0902830
PVSEL 0902740
PCHAS 0926500
PCHAS 0926800 *
PVPCR 0902770
PVLDE 0902790
PCHAS 0925850
PVLDE 0902760
PVSEL 0902720
PVLDE 0902920 *
PVBDA 0902810 *
PVVCY 0902760 *
PVPCR 0902860 *
PVSEL 0902840 *
PY17X 0921200
PY17X 0921300
PY17X 0921000
PY17X 0921100
PBANKA 0919200
PY17X 0920900
PCHAS 0926900
PVVCY 0902770
PVBDA 0902820
PVLDE 0902930
PVSEL 0902850
PVPCR 0902870
P
P
P
P
P
P
P
P
P
P
P
P
P
P
P
P
P
P
P
z-score
-1.3
0
1.1
Species
P. berghei
P. yoeli
P. chabaudi
P. v. vinckei
P. v. brucechwatti
P. v. lentum
P. v. petteri
P. v. subsp.
Maximal gene expression
Low (FPKM < 32)
Medium (32< FPKM < 256)
High (FPKM > 256)

7. haloacid dehalogenase-like hydrolases (*hdh*)

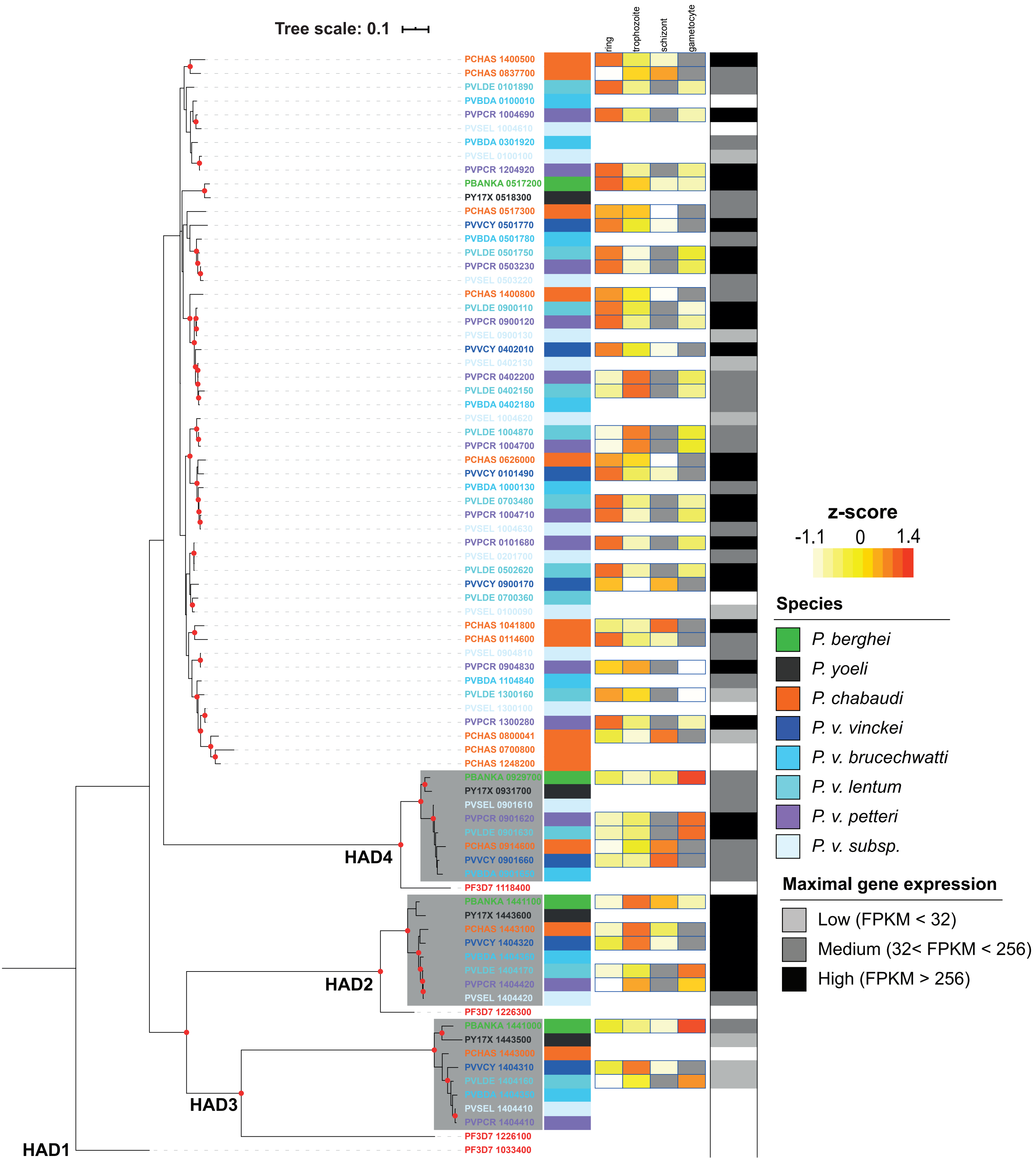

8. lysophospholipases (*lpl*)

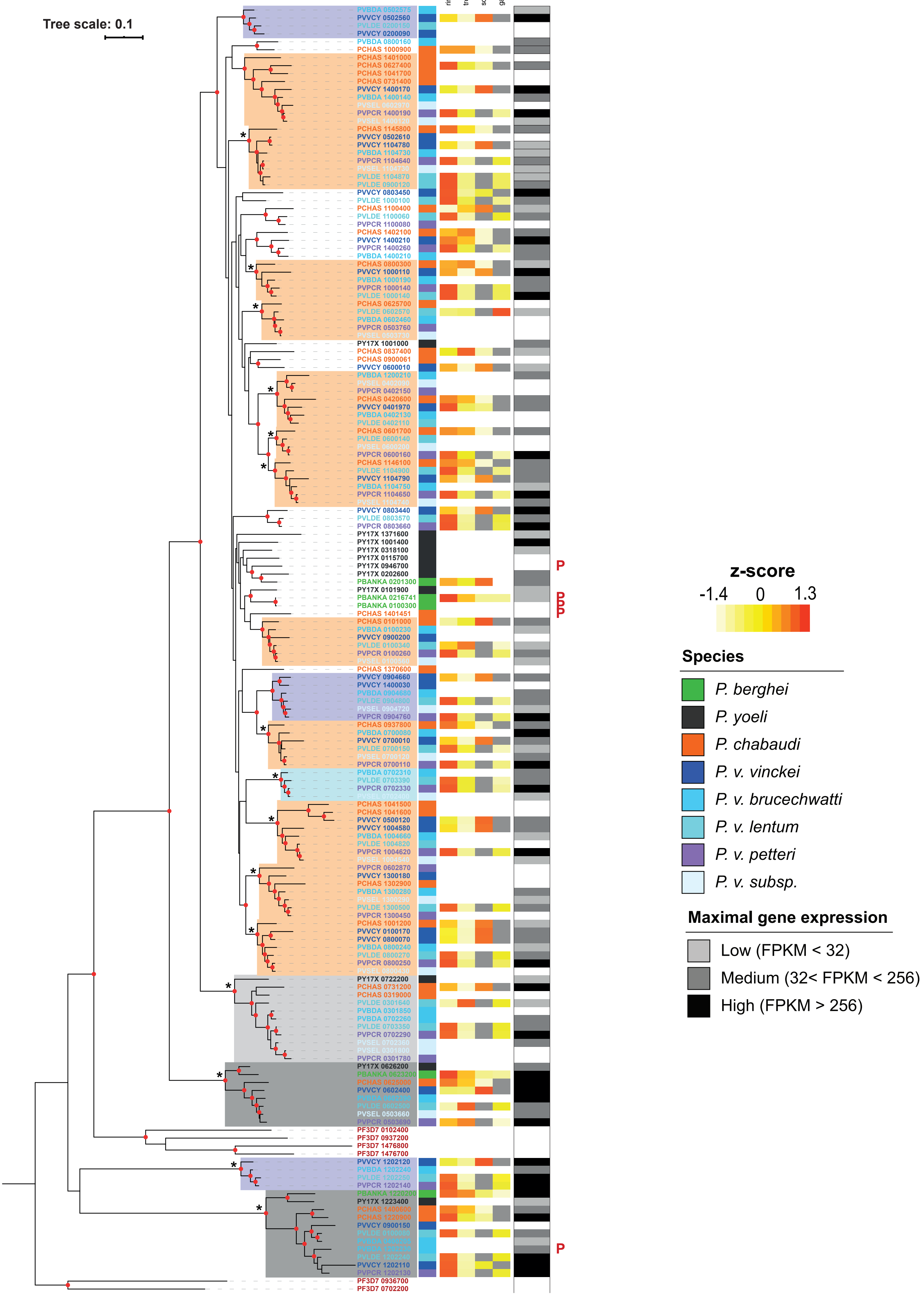

9. reticulocyte binding protein (*p235*)

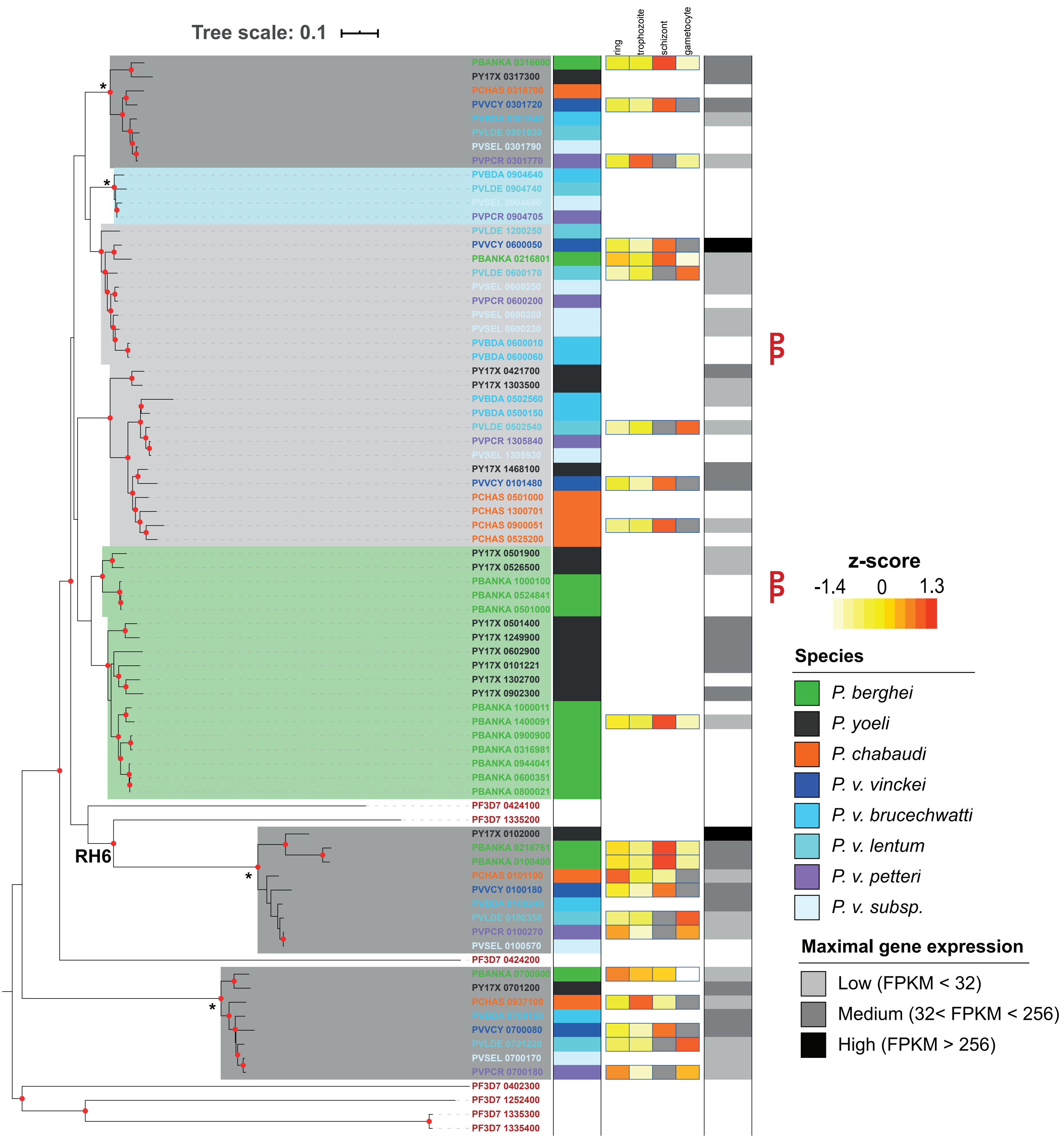

10. *Plasmodium* interspersed repeat proteins (*pir*)

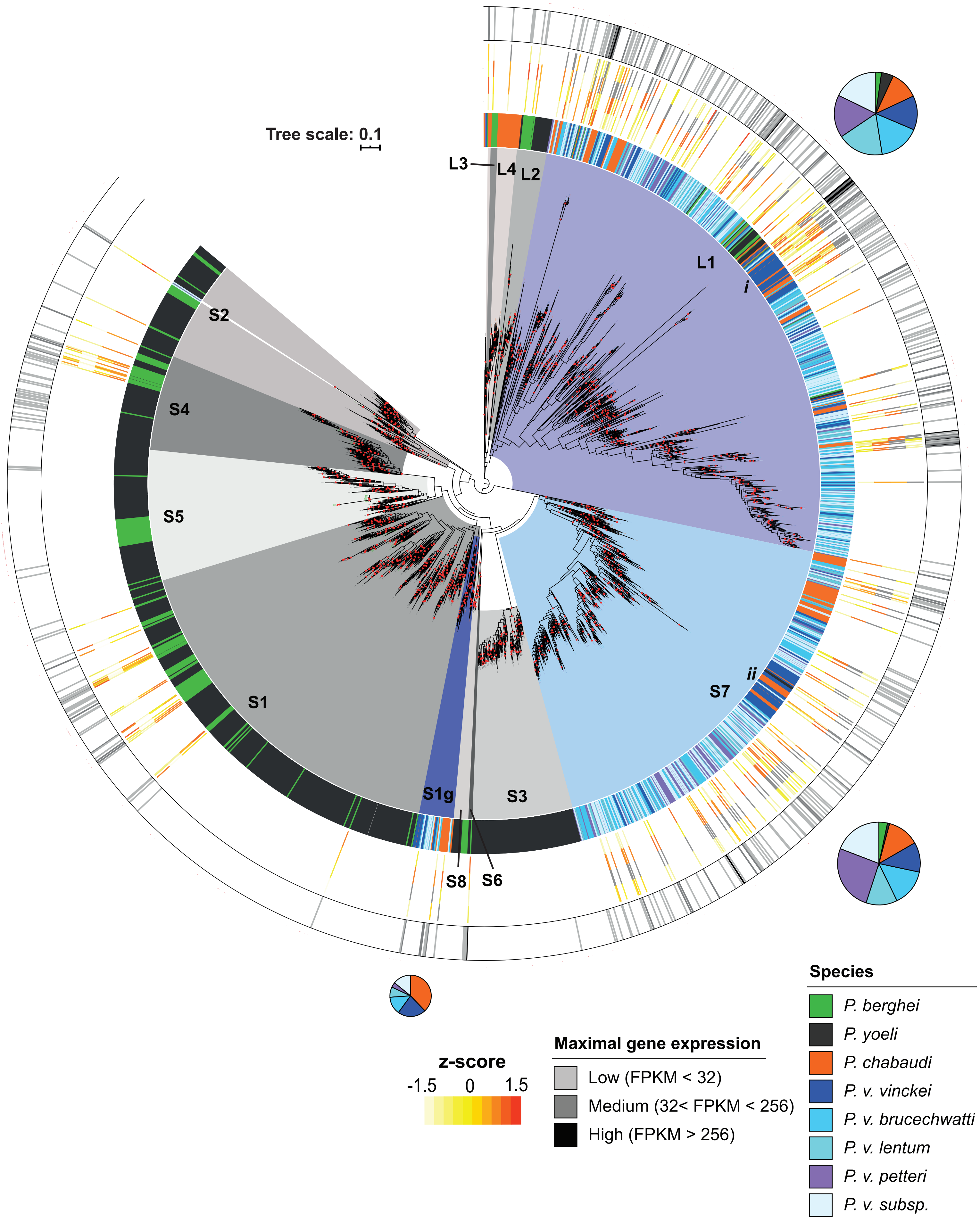

Supplement: Supplementary file 9 — Additional file 9. Maximum Likelihood trees for ten RMP multigene families [file 12915_2021_995_MOESM9_ESM.pdf]
